# Prevalence of dyslipidemia associated with complications in diabetic patients: a nationwide study in Thailand

**DOI:** 10.1186/s12944-019-1034-3

**Published:** 2019-04-06

**Authors:** Ploypun Narindrarangkura, William Bosl, Ram Rangsin, Panadda Hatthachote

**Affiliations:** 1 Department of Military and Community Medicine, Phramongkutklao College of Medicine, 315 Ratchawithi Road, Ratchathewi, Bangkok, 10400 Thailand; 2 Health Informatics Program, School of Nursing and Health Professions of the University of San Francisco, 2130 Fulton St, San Francisco, CA 94117 USA; 3 Department of Physiology, Phramongkutklao College of Medicine, 315 Ratchawithi Road, Ratchathewi, Bangkok, 10400 Thailand

**Keywords:** Prevalence, Dyslipidemia, Diabetes, T2DM, Complications, Thailand

## Abstract

**Background:**

Dyslipidemia is an important modifiable risk factor for cardiovascular disease. It is diagnosed by the presence of an abnormal lipid profile, primarily with elevated levels of plasma cholesterol, triglyceride, or both, or reduced levels of high-density lipoprotein cholesterol. However, some studies have reported increased risk of ischemic stroke with elevated low-density lipoprotein cholesterol (LDL-C) levels and increased risk of cardiovascular mortality independent of LDL-C levels in type 2 diabetes mellitus (T2DM) patients.

**Methods:**

In this cross-sectional study, data were included for Thai adults with diabetes from the Diabetes Mellitus/ Hypertension (DM/HT) study, 2010–2014 (data was collected by the Medical Research Network of the Consortium of Thai Medical Schools). The target population comprised T2DM patients who were treated at a hospital for more than 12 months. Adjusted odds ratios (aORs) and 95% confidence intervals (CIs) were calculated to determine factors associated with dyslipidemia.

**Results:**

In total, 140,557 participants (average age, 60 years) were enrolled, with a dyslipidemia prevalence of 88.9% in the cohort. The factors associated with dyslipidemia included female sex (aOR: 1.47, 95% CI: 1.38–1.56); age < 50 years (aOR: 1.16, 95% CI: 1.10–1.22); waist circumference ≥ 90 cm in males and ≥ 80 cm in females (aOR: 1.23, 95% CI: 1.16–1.31); treatment at a primary care unit (aOR: 1.28, 95% CI: 1.23–1.33); and a history of unknown stroke (aOR: 1.10, 95% CI: 1.02–1.19), coronary revascularization (aOR: 0.85, 95% CI: 0.79–0.91), diabetic nephropathy (aOR: 1.06, 95% CI: 1.01–1.12), or renal insufficiency (aOR: 1.08, 95% CI: 1.02–1.13).

**Conclusions:**

Dyslipidemia is prevalent among Thai T2DMpatients and is associated with gender; age; obesity; central obesity; treatment at a primary care unit; and a history of unknown stroke, coronary revascularization, diabetic nephropathy, and renal insufficiency. Our study results will help increase the awareness of healthcare providers regarding dyslipidemia in diabetic patients. To reduce cardiovascular risk, healthcare professionals should provide regular follow-up and proper advice and ensure primary prevention of vascular complications. Improved education and increased self-awareness regarding the need to change behaviors and regular intake of medication would help decrease dyslipidemia prevalence among diabetic patients.

## Background

Dyslipidemia is a major risk factor for cardiovascular disease, stroke, and type 2 diabetes mellitus (T2DM) [1], but it is modifiable by lifestyle changes and medication [2]. The disorder is characterized by an abnormal lipid profile, which can include elevated levels of plasma cholesterol, triglycerides, or both, or reduced levels of high-density lipoprotein cholesterol (HDL-C) [3, 4]. According to the National Health and Nutrition Examination Survey 2003–2006, lipid abnormalities were present in 53% of US adults [5]. Compared with other middle-income Asian countries, unawareness regarding hypercholesterolemia in Thailand was reportedly the highest at 78% in 2004, with low levels of treatment and control [6]. This was compounded by the results of a national survey in 2009, which found that dyslipidemia was present in 66.5% of the Thai population [7].

Dyslipidemia is one of the risk factors for vascular complications in diabetic patients because it increases free fatty acid flux secondary to insulin resistance and aggravated by increased inflammatory adipokine levels [8]. According to the Framingham Heart Study, in diabetic patients, the prevalence rates for high cholesterol levels were 13% in males and 24% in females, and these rates for high plasma triglyceride levels were 19% in males and 17% in females [9]. A cross-sectional, multicenter, hospital-based diabetes registry conducted in Thailand, covering the year 2003, showed that more than 80% diabetic patients had dyslipidemia, but only 40% patients who received lipid-lowering medications achieved the target low-density lipoprotein cholesterol (LDL-C) levels [10]. In Thailand, socioeconomic development and lifestyle changes such as consumption of Western-style diets, reduced physical activity, and changes in type of work have contributed to the increased dyslipidemia prevalence among the population.

To update ourselves and fill the gap of knowledge, we aimed to determine dyslipidemia prevalence among diabetic patients in Thailand and to assess the association between dyslipidemia and diabetes-related complications.

## Methods

### Study design

In this cross-sectional study, we used secondary data from multicenter, hospital-based diabetes and hypertension registries for the period 2010–2014. This data was collected by the Medical Research Network of the Consortium of Thai Medical Schools, which is responsible for collaboration and networking between medical and public health research in Thailand. We then performed a quantitative analysis to determine dyslipidemia prevalence and associated factors among diabetic patients. The study was reviewed and approved by the Institutional Review Board of the Royal Thai Army Medical Department (S058 h/60_Exp).

### Study population

We used secondary data collected from diabetic patients treated at public Thailand Ministry of Public Health hospitals in Thailand and patients treated at private clinics in Bangkok who participated in the Thailand National Health Security Office program for more than 12 months. Diabetic patients aged ≥18 years who provided signed informed consent were included in the study.

### Data collection

Secondary data were sourced from a database named “An Assessment on Quality of Care among Patients Diagnosed with Type 2 Diabetes and Hypertension Visiting Ministry of Public Health and Bangkok Metropolitan Administration Hospitals in Thailand” (referred to as the Thailand DM/HT study database), for the period 2010–2014. The following participant characteristics were assessed: gender, region, age, body mass index (BMI), waist circumference, health scheme, treatment setting, occupation, and smoking status. We also collected data on lipid profile, fasting plasma glucose, and glycosylated hemoglobin (HbA1c) levels to determine the associated factors. Finally, data on the following complications were included: cerebrovascular accident, cerebral infarction, ischemic stroke, hemorrhagic stroke, unknown stroke, cerebral hemorrhage, transient ischemic attack, angina pectoris, congestive heart failure, myocardial infarction, coronary revascularization, peripheral arterial disease, atrial fibrillation, diabetic retinopathy, diabetic nephropathy, renal insufficiency, and neuropathy.

Dyslipidemia was defined as total cholesterol ≥200 mg/dL, LDL-C ≥ 100 mg/dL, triglycerides ≥150 mg/dL, or HDL-C ≤ 40 mg/dL in males and ≤ 50 mg/dL in females [4, 11]. Furthermore, we separated patients according to six abnormal lipid profiles: pure hypercholesterolemia, pure hypertriglyceridemia, mixed hyperlipidemias, high non-HDL-C, high LDL-C, and low HDL-C.

### Statistical analysis

Secondary data were analyzed using RStudio (Version 1.1.383). The association between variables and dyslipidemia in T2DM Thai patients was assessed using Χ^2^ or Fisher’s exact tests with 95% confidence intervals (CIs). Univariate and multivariate analyses were performed using linear regression modeling and binary logistic regression modeling to determine the independent risk factors for dyslipidemia, with adjusted odds ratios (aORs) and 95% CIs.

## Results

The participants’ characteristics are shown in Table 1. Of the 140,557 participants, 97,627 (69.47%) were female, and the average age was 60 years (four-fifth patients were aged > 50 years). Approximately half of the participants were overweight or obese and 70% had truncal obesity, but most (88.53%) were nonsmokers. Nearly half of the patients received treatment at a primary care unit and most (74.37%) used the universal health coverage system. Dyslipidemia prevalence was 88.92%, and the main type of abnormal lipid profile was a low HDL-C (59.59%) (Table 2). The mean lipid levels among T2DM patients are summarized in Table 3, which shows that there were no significant differences regarding lipid levels among the patients per year. On separating the mean lipid, fasting blood sugar, and HbA1c levels by gender, age, BMI, waist circumference, and smoking subgroups, we found that female sex and obesity were associated with higher mean levels in each lipid profile category (Table 4).

**Table 1:** Diabetic patients’ characteristic and dyslipidemia prevalence (*N* = 140,557)

	n	%	Dyslipidemia	
			n	%
Gender				
Male	42,902	30.53	26,551	84.62
Female	97,627	69.47	65,107	90.80
Region				
North	28,984	20.62	19,893	89.11
Central	32,611	23.2	18,170	85.85
East	12,753	9.07	7160	85.96
North-East	47,756	33.98	32,892	91.74
South	9757	6.94	7135	87.40
South Boundary	8696	6.19	6408	88.51
Age (years)*	60.31 ± 10.79 (18–99)			
< 50	22,626	16.11	15,055	91.12
≥ 50	117,853	83.89	76,562	88.50
Body mass index (kg/m^2^) **				
< 18.50	4736	3.6	2853	82.86
18.5–22.9	33,806	25.69	21,918	87.65
23.0–24.9	27,105	20.6	18,135	89.77
25.0–29.9	47,290	35.93	31,632	90.19
≥ 30.0	18,671	14.19	12,466	89.83
Waist circumference (cm)				
Male < 90, Female < 80	12,233	28.76	8131	84.55
Male ≥90, Female ≥80	30,309	71.24	21,469	89.29
Health scheme				
Universal Health Coverage	103,628	74.37	68,172	90.24
Government	23,788	17.07	15,177	83.43
Social Security	4499	3.23	2812	86.07
State Enterprise Officer	501	0.36	314	83.96
Cash	638	0.46	334	85.64
Other	6278	4.51	4101	92.12
Hospital setting				
Tertiary Care Hospital	40,040	30.04	27,294	87.58
Secondary Care Hospital	30,460	22.85	19,334	87.25
Primary Care Unit	62,806	47.11	42,051	91.02
Occupation				
Agriculture	54,245	39.14	37,305	91.45
Non-agriculture	72,707	52.46	46,393	87.74
Unemployment	7557	5.45	4847	83.87
Others	4081	2.94	2106	85.23
Smoking				
Non-smoker	108,340	88.53	72,923	89.27
Ex-smoker	8608	7.03	5561	86.15
Current smoker	5434	4.44	3595	87.05

*Mean ± SD (min–max)

**WHO/IASO/IOTF. The Asia-Pacific perspective: Redefining obesity and its treatment. Health Communications Australia: Melbourne, 2000

**Table 2:** Prevalence of abnormal lipid profile among T2DM Thai patients in 2010–2014

	n	%
Dyslipidemia*	91,658	88.92
Pure hypercholesterolemia	39,738	35.05
Pure hypertriglyceridemia	59,151	49.94
Mixed hyperlipidemias	25,201	21.72
High non-HDL	55,255	53.41
High LDL	66,508	56.54
Low HDL	63,914	59.59

*Defined by total cholesterol ≥200 mg/dL, LDL ≥ 100 mg/dL, TG ≥ 150 mg/dL, or HDL ≤ 40 mg/dL in males and ≤ 50 mg/dL in females

**Table 3:** Mean ± SD for lipid profile among T2DM Thai patients in 2010–2014

	2010	2011	2012	2013	2014	Total	*p* for trend
Total cholesterol	188.62 ± 51.19	187.8 ± 45.47	187.44 ± 45.65	187.56 ± 44.5	188.59 ± 46.53	188 ± 46.57	0.8065
Triglyceride	177.76 ± 118.67	174.46 ± 93.48	175.04 ± 104.47	175.34 ± 111.52	175.62 ± 111.91	175.57 ± 108.23	0.7071
HDL	46.06 ± 27.39	45.14 ± 12.46	45.82 ± 13.02	46.74 ± 13.88	47.20 ± 15.53	46.25 ± 16.87	0.2597
LDL	110.61 ± 42.67	109.33 ± 36.77	109.62 ± 37.47	108.61 ± 37.29	109.33 ± 37.95	109.45 ± 38.3	0.5661

**Table 4:** Mean lipid and fasting blood sugar levels among Thai patients with T2DM in 2010–2014

	Total Cholesterol	Triglyceride	HDL	LDL	FBS	HbA1c
Gender						
Male	181.68	174.92	43.8	105.96	150.9	7.85
Female	190.78	175.86	47.32	110.97	153.76	8.08
Age (years)						
< 50	188.63	183.49	45.83	110.28	166.13	8.49
≥ 50	187.89	174.06	46.32	109.29	150.37	7.92
Body mass index (kg/m^2^) **						
< 30	187.88	175.9	46.07	109.39	152.58	8.02
≥ 30	189.5	177.85	46.61	110.48	156.51	8.07
Waist circumference (cm)**						
Male < 90, Female < 80	184.1	165.28	47.15	106.49	154.2	7.98
Male ≥90, Female ≥80	189.56	181.45	46.72	109.71	156.19	8.06
Smoking						
Current smoker	184.33	185.87	43.72	106.92	156.52	7.96
Ex-smoker	181.87	175.61	43.5	105.52	151.84	7.94
Non-smoker	188.66	174.85	46.64	109.83	153.05	8.01

**WHO/IASO/IOTF. The Asia-Pacific perspective: Redefining obesity and its treatment. Health Communications Australia: Melbourne, 2000

Univariate and multivariate analyses were performed to determine the factors associated with lipid profiles and fasting plasma glucose levels (Table 5). Linear regression modeling revealed that total cholesterol (aOR: 1.07, 95% CI: 1.05–1.08), triglycerides (aOR: 1.06, 95% CI: 1.05–1.06), and LDL-C (aOR: 1.05, 95% CI: 1.04–1.07) were associated with fasting plasma glucose levels. To determine the factors associated with dyslipidemia, logistic regression modeling was used with univariate and multivariate analyses. As shown in Table 6, the independent factors associated with dyslipidemia were gender, age, waist circumference, unknown stroke, coronary revascularization, diabetic nephropathy, and renal insufficiency.

**Table 5:** Univariate and multivariate of lipid profile and fasting plasma glucose in T2DM Thai patients

	Total	Fasting plasma glucose level (mg/dl) *	Crude odds ratio 95% CI	*p*-value	Adjusted odds ratio 95% CI	*p*-value
Total cholesterol	102,792	152.8 ± 53.6 (1–835)	1.15 (1.14–0.16)	< 0.001	1.07 (1.05–1.08)	< 0.001
Triglyceride	107,376	152.6 ± 53.4 (1–835)	1.07 (1.06–1.07)	< 0.001	1.06 (1.05–1.06)	< 0.001
HDL	97,450	152.7 ± 53.6 (1–835)	0.99 (0.97–1.01)	0.155		
LDL	106,699	152.5 ± 53.2 (1–835)	1.13 (1.13–1.14)	< 0.001	1.05 (1.04–1.07)	< 0.001
Non-HDL	96,598	152.7 ± 53.5 (1–835)	1.16 (1.15–1.17)	< 0.001		

*Mean ± SD (Min–Max)

Linear Regression; After adjusted for HDL and non-HDL level

**Table 6:** Univariate and multivariate analysis of associated factors and dyslipidemia in T2DM Thai patients

	Total	Dyslipidemia		Adjusted odds ratio 95% CI	*p*-value
		n	%		
Gender					
Male	42,902	26,551	84.62	1	
Female	97,627	65,107	90.80	1.47 (1.38–1.56)	< 0.001
Age (years)*	60.31 ± 10.79 (18–99)				
≥ 50	117,853	76,562	88.50	1	
< 50	22,626	15,055	91.12	1.16 (1.10–1.22)	< 0.001
Body mass index (kg/m^2^) **					
< 18.50	4736	2853	82.86	1	
18.5–22.9	33,806	21,918	87.65	1.97 (1.80–2.15)	< 0.001
23.0–24.9	27,105	18,135	89.77	1.51 (1.39–1.64)	< 0.001
25.0–29.9	47,290	31,632	90.19	1.92 (1.77–2.09)	< 0.001
≥ 30.0	18,671	12,466	89.83	2.03 (1.87–2.20)	< 0.001
Waist circumference (cm)					
Male < 90, Female < 80	12,233	8131	84.55	1	
Male ≥90, Female ≥80	30,309	21,469	89.29	1.23 (1.16–1.31)	< 0.001
Hospital setting					
Tertiary Care Hospital	40,040	27,294	87.58	1	
Secondary Care Hospital	30,460	19,334	87.25	0.99 (0.94–1.03)	0.583
Primary Care Unit	62,806	42,051	91.02	1.28 (1.23–1.33)	< 0.001
Smoking					
Non-smoker	108,340	72,923	89.27	1	
Ex-smoker	8608	5561	86.15	1.12 (1.05–1.20)	< 0.001
Current smoker	5434	3595	87.05	1.23 (1.13–1.33)	< 0.001
Complications					
Unknown stroke					
No	63,781	42,384	87.86	1	
Yes	641	399	85.81	1.1 (1.02–1.19)	0.011
Coronary revascularization					
No	66,791	44,764	88.16	1	
Yes	301	188	82.10	0.85 (0.79–0.91)	< 0.001
Diabetic nephropathy					
No	72,468	49,062	88.21	1	
Yes	9325	6443	90.00	1.06 (1.01–1.12)	0.031
Renal insufficiency					
No	68,058	45,879	88.03	1	
Yes	12,957	8780	90.22	1.08 (1.02–1.13)	0.005

Multiple logistic regression; using Multivariate Imputation by Chained Equations, Both direction

After adjusted for cerebrovascular accident, cerebral infarction, hemorrhagic stroke, myocardial infarction, and neuropathy

We obtained several noteworthy results. Females had 1.47-time increased odds of having dyslipidemia over males (*p* < 0.001), while patients aged < 50 years had a 1.16-time increased odds over those aged > 50 years (*p* < 0.001). Patients with a higher BMI had higher odds of having a disease, with obesity being associated with 2.03-time increased odds over underweight (*p* < 0.001). Men with a waist circumference ≥ 90 cm and women with a waist circumference ≥ 80 cm had 1.23-time increased odds of having dyslipidemia (*p* < 0.001). Interestingly, receiving treatment at a primary care unit was associated with 1.28-time increased odds of having dyslipidemia over receiving treatment at a tertiary care unit (*p* < 0.001). Being a current or ex-smoker was associated with 1.23-time (*p* < 0.001) or 1.12-time (*p* < 0.001) increased odds of having dyslipidemia, respectively. Furthermore, we found associations between dyslipidemia and complications, notably for a history of stroke (aOR 1.1, *p* = 0.011) and coronary revascularization (aOR 0.85, *p* < 0.001) and for patients with diabetic nephropathy (aOR 1.06, *p* = 0.031) and renal insufficiency (aOR 1.08, *p* = 0.005).

## Discussion

In this study, based on the revised National Cholesterol Education Program-Adult Treatment Panel III criteria, we aimed to determine dyslipidemia prevalence among patients visiting hospitals for diabetes throughout Thailand. The last comparable study was conducted between April and December 2003 only in the diabetes clinics of tertiary care hospitals. Therefore, we anticipated that this study would update our knowledge regarding dyslipidemia and the associated outcomes among T2DM patients in Thailand.

There was an obvious high dyslipidemia prevalence among Thai patients with diabetes (88.9%), which had remained largely unchanged compared with that reported in 2003 (80%) [10, 12]. In a similar report from China, dyslipidemia prevalence was 67.1% among Chinese T2DM patients [13]. Likewise, according to the United States National Health and Nutritional Examination Survey for 1999–2000, the prevalence of LDL-C > 100 mg/dL was 71% among males and 78.9% among females with diabetes, indicating that dyslipidemia control was inadequate based on the American Diabetes Association guidelines [14]. The reasons for high dyslipidemia prevalence in these patients can be explained in several ways.

Healthcare system as well as appropriate patient education plays an important role in disease control. According to the Thai report in 2003, medications were not taken by 30% diabetic patients who fulfilled the criteria for receiving lipid-lowering medications, and only 40.1% of those who took their medications achieved the target LDL levels of < 100 mg/dL [11]. Furthermore, it is noteworthy that patients covered by government-supported health plan were less likely to receive lipid-lowering medications compared with those covered by private health plans. The impact of shifting toward a modern lifestyle should not be underestimated, with developing countries increasingly adopting Western-style diets that include high-calorie foods with increased carbohydrate, fat, and red meat content and low fiber content. These dietary changes correlate with a rapid growth in the prevalence of obesity, metabolic syndrome, and T2DM [15]. The Thai government, therefore, launched a plan named “Thailand Healthy Lifestyle Strategy 2011–2020 Plan” that aims to decrease the prevalence of and complications, disability, mortality, and cost of illness associated with major diseases, including diabetes, hypertension, ischemic heart disease, stroke, and cancer [16]. However, despite a good initiation process, this plan has not resulted in any significant changes in the prevalence of the abovementioned non-communicable diseases [17].

When we focused on the subtypes of dyslipidemia, the major types found were low HDL levels (59.6%), followed by high LDL (56.5%) and non-HDL (53.4%) levels and pure hypertriglyceridemia (49.9%), with lower rates of pure hypercholesterolemia (35.1%) and mixed hyperlipidemias (21.7%). Similar to these results, studies in China and other Asian countries have shown hypertriglyceridemia and low HDL-C levels to be the major types of dyslipidemia [18–22]. Conversely, in the US population, the major type of abnormal lipid profile is high LDL levels (27%) [5].

Furthermore, we found multiple factors to be associated with dyslipidemia among Thai diabetic patients. Interestingly, age < 50 years was significantly associated with dyslipidemia in diabetic patients, which could be attributed to work pressure and a lack of physical activity [2]. As expected, obesity was significantly associated with dyslipidemia in T2DM patients. In a comparable study from India, it was reported that T2DM pa tients who had obesity along with hypertriglyceridemia and high LDL-C and non-HDL-C levels exhibited poorly controlled HbA1c levels compared with a group with normal lipid profiles [23]. Moreover, several reports have shown a significant influence of lipid levels on HbA1c levels and cardiovascular complications, possibly as a consequence of increased insulin resistance [24–26].

In Thailand, hospitals are classified into three categories: regional hospitals (tertiary care), general hospitals (secondary care), and community hospitals (primary care). We found that treatment at a primary care unit was significantly associated with dyslipidemia in T2DM patients. This finding may reflect the fact that primary care units are located at the district level and are limited to providing only primary care treatment. Patients with uncomplicated diseases are referred back to primary care units (which have no specialists) to receive regular medications. General practitioners might, therefore, underdiagnose the complications because of lack of experience and resources (including some laboratory tests). Furthermore, primary care units have limited available drug options and often lack access to advanced or novel therapies. A survey of primary care settings in Thailand showed that necessary and routine aspects of diabetic care were not regularly performed by healthcare providers [27].

Researchers have consistently found an association between dyslipidemia and smoking [28–30]. Our results also confirmed that current smoking was significantly related to dyslipidemia prevalence in diabetic patients. In a community-based prospective cohort of American Indians, it was shown that hypertriglyceridemia and low HDL-C levels among diabetic patients were associated with a 2.13-fold greater hazard ratio for stroke (95% CI, 1.06–4.29) [31].

We also found clear associations between certain vascular complications and dyslipidemia in Thai T2DM patients. For instance, a significant association was found between dyslipidemia and stroke. The mechanism underlying the increased cardiovascular morbidity and mortality could be explained by factors such as increased leptin, adipocyte dysregulation, increased insulin resistance, and increased C-reactive protein levels [32]. We also showed a significant association between dyslipidemia and renal diseases (diabetic nephropathy and renal insufficiency), consistent with other studies reporting a significant association of dyslipidemia and microalbuminuria with the risk of cardiovascular and kidney complications in diabetic patients [33]. However, a history of coronary revascularization was found to be protective against dyslipidemia, probably because of the closer attention paid to these patients for the control of hypertension, cholesterol, and HbA1c, as well as assistance with smoking cessation [34]. Dyslipidemia is a risk factor that can be modified with lifestyle interventions, and each of the abovementioned vascular complications can be targeted by primary prevention as well as by appropriate secondary prevention.

## Conclusion

In summary, based on secondary data from hospitals throughout Thailand, we showed high dyslipidemia prevalence in Thai diabetic patients. Dyslipidemia in this patient population was associated with gender, age, obesity, central obesity, treatment at a primary care unit, and several vascular complications. Our findings should raise awareness regarding the need for healthcare providers to offer better management of dyslipidemia in Thai diabetic patients. Indeed, regular follow-up, proper advice, and greater attention to the primary prevention of vascular complications could reduce the risk of cardiovascular diseases. Specific efforts to educate patients and increase their awareness regarding the need to change behaviors and regularly take medication would be a positive step toward decreasing dyslipidemia prevalence in diabetic patients.

## Abbreviations

aOR: adjusted odds ratio
BMI: body mass index
CI: confidence interval
HDL-C: high-density lipoprotein cholesterol
LDL-C: low-density lipoprotein cholesterol
T2DM: type 2 diabetes mellitus
